# Mortality in patients with psoriatic arthritis: a systematic review and meta-analysis

**DOI:** 10.3389/fimmu.2025.1622159

**Published:** 2025-08-18

**Authors:** Hong Huang, Wenhui Xie, Yan Geng, Yong Fan, Zhuoli Zhang

**Affiliations:** Department of Rheumatology and Clinical Immunology, Peking University First Hospital, Beijing, China

**Keywords:** psoriatic arthritis, systematic review, meta-analysis, mortality, causes of mortality

## Abstract

**Objective:**

The debate persists regarding whether patients with psoriatic arthritis (PsA) face an increased risk of mortality. We aimed to ascertain the magnitude of all-cause mortality risk in patients with PsA compared to the general population through a systematic review and meta-analysis.

**Methods:**

We conducted a comprehensive search of PubMed, EMBASE, and the Cochrane Library for studies published from inception to June 2025. STATA meta-analysis software was used to calculate the pooled risk estimates for mortality, represented as the standardized mortality ratio (SMR).

**Results:**

Among the 4,502 articles identified in our research, 20 studies were included in the analysis. Overall, our findings revealed a 1.12-fold increased risk of death among patients with PsA compared to the general population (meta-SMR: 1.12, 95% CI 1.09-1.15). Subgroup analyses showed that mortality risks were elevated in Asian countries (meta-SMR: 1.28, 95% CI 1.04-1.57), in population-based studies (meta-SMR: 1.13, 95% CI 1.02-1.25), and among studies including over 1,000 patients (meta-SMR: 1.12, 95% CI 1.01-1.25). Malignancy, cardiovascular and cerebrovascular diseases, and infection/respiratory diseases emerged as the most frequent causes of mortality.

**Conclusion:**

Our analysis suggested modestly elevated mortality in patients with PsA compared to the general population, though heterogeneity warrants cautious interpretation. Malignancy, cardiovascular and cerebrovascular diseases, and infection/respiratory disease were frequent causes of mortality and warrant further investigation.

**Systematic review registration:**

https://www.crd.york.ac.uk/prospero/, identifier CRD42021275209.

## Introduction

Psoriatic arthritis (PsA) is a chronic inflammatory disease associated with psoriasis (1). It is characterized by diverse clinical features, including enthesitis, arthritis, dactylitis, and axial involvement. In addition to musculoskeletal manifestations, PsA is frequently associated with obesity, type 2 diabetes, hypertension, and metabolic syndrome (2). Nevertheless, whether patients with PsA face a heightened risk of mortality compared to the general population remains debated.

While numerous studies have investigated the prognosis of PsA, their findings remain inconsistent. A cohort study of 9,572 patients with PsA documented 682 deaths (7.1%) during a median follow-up of 6.46 years, revealing an adjusted standardized mortality ratio (SMR) of 1.47 versus general population controls (3). Several other studies reported similar findings, with SMRs ranging from 1.11 to 2.19 (4–9). Conversely, publications from other regions have found no significant association between PsA and increased mortality rates (10–12). A previous meta-analysis of 11 studies indicated that all-cause mortality was not increased among patients with PsA (13). However, several recent studies with large sample sizes have presented conflicting findings, prompting a re-evaluation of this important issue (9, 14–18). This meta-analysis aimed to quantify the risk of mortality in patients with PsA through systematic synthesis of observational studies.

This article is a revised and expanded version of a paper presented at the 69th Annual Scientific Meeting of the Japan College of Rheumatology (JCR 2025), held in Fukuoka, Japan (19).

## Methods

This systematic review and meta-analysis adhered to the Preferred Reporting Items for Systematic Reviews and Meta-Analyses (PRISMA) guidelines (20), with a prospectively registered protocol (registration number: PROSPERO CRD42021275209). Three independent investigators (HH, WX, and YF) performed literature screening, data extraction, and quality assessment.

### Strategies of literature search and inclusion criteria

PubMed, EMBASE, and the Cochrane Library were systematically searched through June 2025 using comprehensive terms for “*psoriatic arthritis*” and “*mortality*,” without date/language restrictions. We also searched major annual meetings—specifically the annual meetings of the American College of Rheumatology (ACR) and the European Congress of Rheumatology (EULAR)—from 2016 to 2024. Supplementary hand-searching included screening the reference lists of relevant reviews and eligible articles. No language restrictions were applied.

We included only population-based and cohort studies that reported all-cause mortality rates among patients with PsA and either compared these rates with those of the general population or matched controls, or directly reported the SMR.

In instances where duplicate data appeared in different studies, preference was given to the most up-to-date data. After eliminating duplicates, two independent researchers (HH and WX) conducted a thorough screening of all titles and abstracts, followed by a comprehensive evaluation of the complete texts of potentially relevant articles. Case reports, case series, reviews, commentaries, and editorials were excluded. The detailed search protocol is available in **Supplementary Appendix S1**.

### Data extraction and quality assessment

Two investigators (HH and FY) independently performed data extraction using a standardized template. Extracted data included study characteristics such as authorship, publication year, geographic origin, research design, clinical setting, date source, enrollment period, and follow-up duration; cohort attributes such as sample size, sex distribution, mean age, diagnostic criteria, clinical manifestations, and serological parameters; and outcomes including all-cause mortality rates and reported mortality risk factors.

Discrepancies in data extraction were adjudicated by a third investigator (ZZ). Two independent assessors evaluated study quality using the Newcastle–Ottawa Scale (NOS), with quality stratification defined as: high (7–9 stars), moderate (4–6 stars), or low (0–3 stars) (21).

### Data synthesis and statistical analysis

Meta-analytic computations were conducted using Stata version 15.1 (StataCorp LP), employing DerSimonian–Laird random-effects models to derive SMR with 95% confidence intervals (CIs). Statistical significance was determined using two-sided tests with α=0.05. Gender-stratified meta-SMRs were calculated for male and female subgroups.

Heterogeneity was quantified using the I² statistic and interpreted as low (≤25%), moderate (26%–50%), or substantial (>50%). Sources of heterogeneity were explored through predefined subgroup meta-analyses and sensitivity assessments. All included studies underwent prespecified stratification by sex, study design (prospective/retrospective cohort), geographic region, population setting (population-based vs. tertiary-care referral cohorts), and cohort size (≥1,000 vs. <1,000 patients). The primary outcome was all-cause mortality, expressed as SMR among patients with PsA relative to the general population.

Sensitivity analysis was performed using a leave-one-out meta-analysis to assess the influence of individual studies on the pooled estimates and to evaluate result robustness (22). Mortality risk factors in PsA were analyzed by pooling extracted risk estimates (95% CIs) using fixed-effects models. Given the low frequency of events, hazard ratios (HRs) were treated as risk ratios (RRs) to ensure consistency of effect size metrics. Forest plots were used to visualize pooled estimates, while funnel plot inspection and Egger’s/Begg’s tests quantitatively evaluated small-study effects (23).

## Results

### Study selection, characteristics, and quality assessment

**Figure 1:**
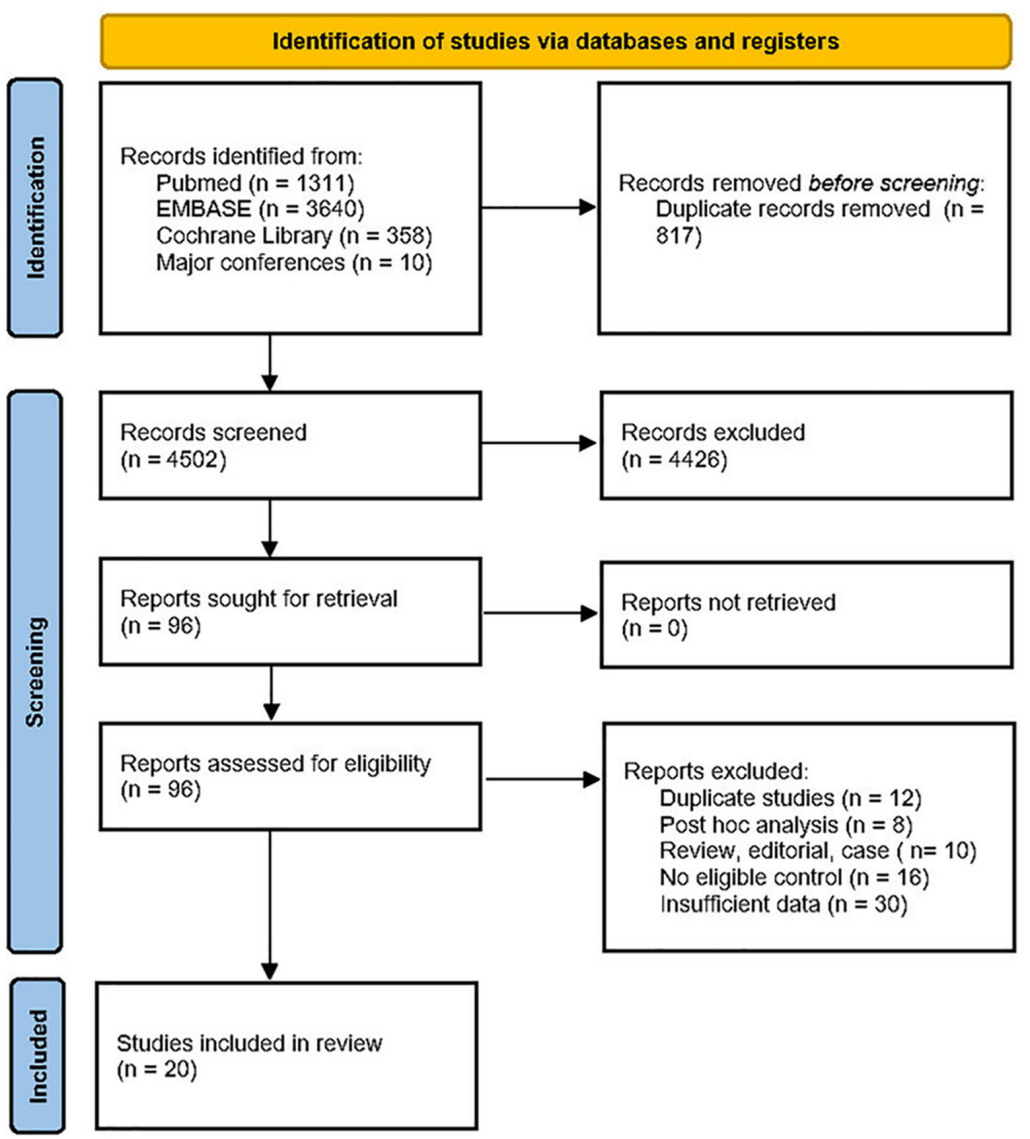
PRISMA flowchart of study selection.

Initially, 4,502 articles were identified through the comprehensive search. After removing duplicates and screening titles and abstracts, 96 full-text articles were thoroughly assessed for eligibility. Ultimately, 20 studies involving more than 130,000 patients were deemed eligible for data extraction and analysis (3–12, 14–18, 24–28). The flowchart illustrating the study selection process is shown in **Figure 1**. 

A summary of the main characteristics of the included studies is provided in **Table 1**. Of the 20 studies, 9 were conducted in European countries (4, 9–11, 14, 17, 25–27), 4 in North America (8, 12, 24, 28) and 7 in Asian countries (3, 5–7, 15, 16, 18). The mean age of patients at study entry ranged from 45 to 59 years. The proportion of female patients ranged from 39% to 66%.

**Table 1:** Main characteristics of the included studies.

No.	Author, year of publication	Region	Study design	Setting	Time period	No. patients	No. Females	Age at study entry (y) (Mean/median)	Death events, n
1	Wilson FC et al., 2009 (23)	Minnesota, USA	retrospective cohort	population-based	1969-1999	147	57	NA	NA
2	Buckley C et al., 2010 (24)	Bath, UK	retrospective cohort	single center	1985-2007	453	221	49	37
3	Ahlehoff O et al., 2011 (4)	Denmark	retrospective cohort	population-based	1997-2006	607	NA	NA	
4	Mok CC et al., 2011 (5)	Hong Kong, China	retrospective cohort	population-based	1999-2008	778	354	52	51
5	Love TJ et al., 2013 (25)	Iceland	retrospective cohort	population-based	1971-2003	293	186	NA	60
6	Ogdie A et al., 2014 (10)	UK	cohort study	population-based	1994-2010	8706	4266	NA	470
7	Juneblad K et al., 2016 (26)	Västerbotten, Sweden	retrospective cohort	referral cohort	1995-2005	464	230	59.5	44
8	Lee MS et al., 2017 (3)	Taiwan, China	retrospective cohort	population-based	2001-2012	9572	NA	NA	682
9	Cheung TT et al., 2018 (6)	Hong Kong, China	cohort study	NA	1997-2016	2165	NA	NA	187
10	Dai XY et al., 2018 (7)	Taiwan, China	cohort study	population-based	2000-2001	8795	3561	45	573
11	Skov L et al., 2019 (11)	Denmark	observational cohort	population-based	1998-2014	9817	5771	50	764
12	Elalouf O et al., 2020 (12)	Toronto, Canada	prospective cohort	single center	1978-2017	1490	660	45	225
13	Colaco K et al., 2021 (8)	Ontario, Canada	retrospective cohort	population-based	1996-2016	15430	NA	NA	221
14	Karmacharya P et al., 2021 (27)	Minnesota, USA	retrospective cohort	population-based	2000-2017	164	77	46	40
15	Bournia VK et al., 2021 (14)	Greece	retrospective cohort	population-based	2015-2019	13779	7565	54	376
16	Haddad A, et al., 2022 (15)	Israeli	retrospective cohort	population-based	2003-2018	5275	2807	52	471
17	Iskandar IYK et al., 2022 (16)	Taiwan, China	retrospective cohort	population-based	2006-2017	NA	NA	NA	
18	Kerola AM et al., 2022 (17)	Norway	cohort study	population-based	2008-2017	18700	9991	49.98	911
19	Erden A et al., 2023 (18)	Turkey	prospective cohort	multicenter	2014-2020	1185	780	NA	31
20	Exarchou S et al., 2023 (9)	Sweden	cohort study	population-based	2001-2017	33026	18022	52	3121

### Mortality in PsA patients

**Figure 2:**
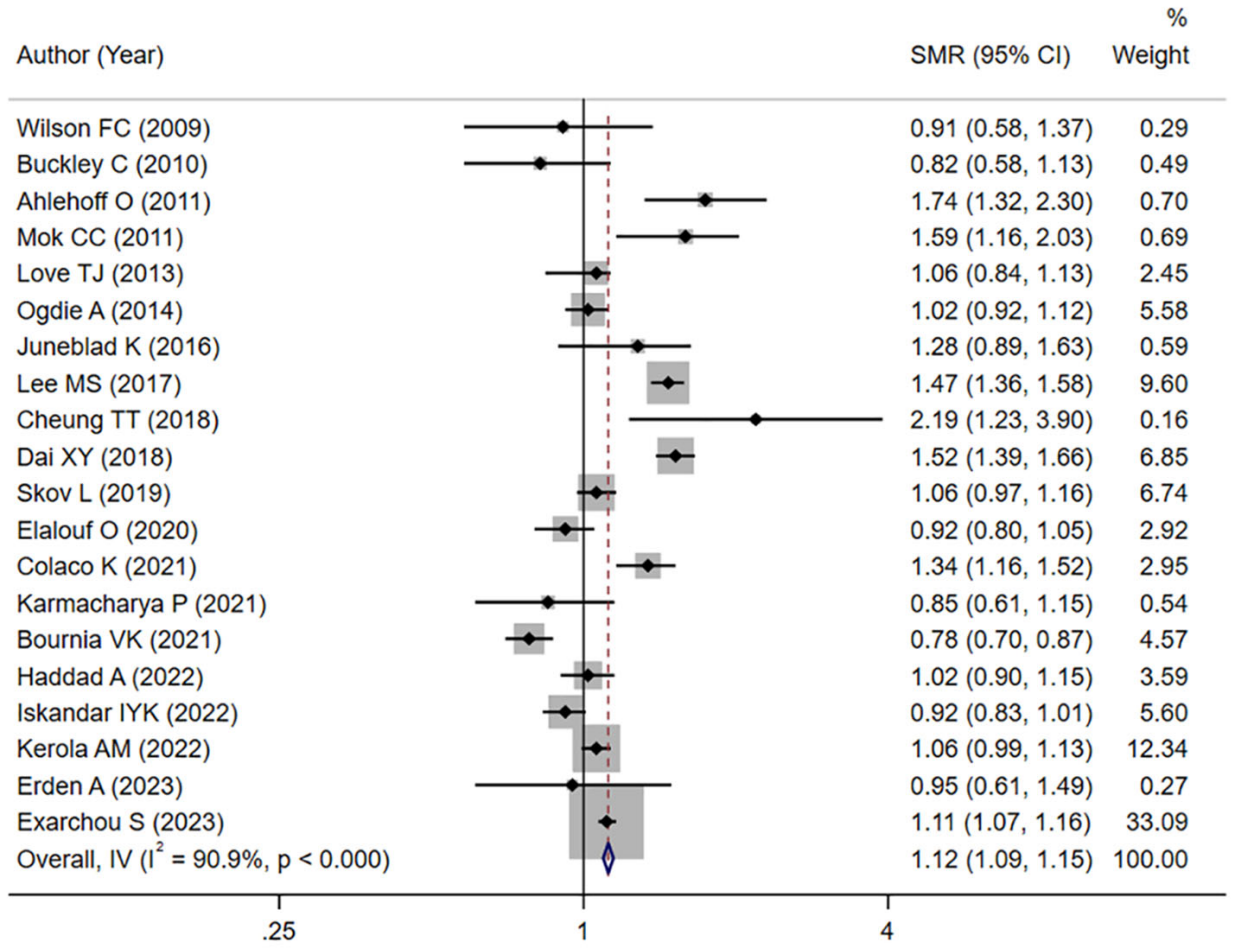
Standardized mortality ratio in patients with PsA.

Overall, the mortality hazard among patients with PsA exhibited a significant elevation compared to the general population (meta-SMR: 1.12; 95% CI: 1.09–1.15), with substantial heterogeneity across the included studies (I^2^ = 90.9%, **Figure 2**).

Seven studies reported sex-specific mortality estimates. A higher summary meta-SMR was observed among female patients (meta-SMR: 1.18; 95% CI: 1.06–1.31; I²=63.4%), while no significant difference was observed among male patients (meta-SMR: 1.01; 95% CI: 0.82–1.25; I²=68.5%) (**Supplementary Figure S1**).

### Subgroup analysis

Subgroup analyses revealed that multiple factors may influence mortality risk. Elevated meta-SMRs were observed in studies from Asian countries (meta-SMR: 1.28; 95% CI: 1.04–1.57), population-based studies (meta-SMR: 1.13; 95% CI: 1.02–1.25), and studies involving more than 1,000 patients (meta-SMR: 1.12; 95% CI: 1.01–1.25). Notably, heterogeneity was reduced in analyses stratified by geographic region, study setting, and cohort size (**Table 2** and **Supplementary Figure S2**).

**Table 2:** Subgroup analyses of mortality in patients with PsA.

Subgroups (No. of studies)	Meta-SMR (95% CI)	I^2^, %
Region		
North America (4)	1.01 (0.78, 1.31)	83.3
Europe (9)	1.05 (0.96, 1.15)	84.7
Asia (7)	1.28 (1.04, 1.57)	93.4
Setting		
Population-based (15)	1.13 (1.02, 1.25)	92.7
Referral cohorts (4)	0.97 (0.82, 1.15)	36.0
Number of patients		
< 1000 (7)	1.15 (0.93, 1.42)	74.9
> 1000 (12)	1.12 (1.01, 1.25)	93.5

### Study quality assessment analysis and sensitivity

The median NOS score among the included studies was 7 (range: 5–8) (**Supplementary Table S1**). A study from Hong Kong involving more severe patients reported a particularly high SMR (meta-SMR: 2.19; 95% CI: 1.23–3.90) (6). When this study was excluded, the pooled meta-SMR was changed to 1.10 (95% CI: 1.01–1.21), with a corresponding I^2^ of 91.2%.

### Predictors of mortality and causes of mortality

Risk factors associated with mortality were analyzed using demographic, clinical, and laboratory data from five studies with available data (**Supplementary Table S2**) (9, 12, 15, 18, 27). Individuals characterized by older age, male sex, elevated acute-phase reactants, and the presence of comorbidities appeared to be associated with increased mortality risk. However, due to limited data, meta-analysis of these predictors was not feasible.

A total of 11 studies reported the etiology of mortality among 6,063 individuals diagnosed with PsA. Malignancy (2,023 patients; 33.4%), cardiovascular and cerebrovascular diseases (1,743 patients; 28.8%), and infection/respiratory disease (543 patients; 9.0%) were the most frequent causes of death (**Supplementary Table S2**).

### Publication bias

**Figure 3:**
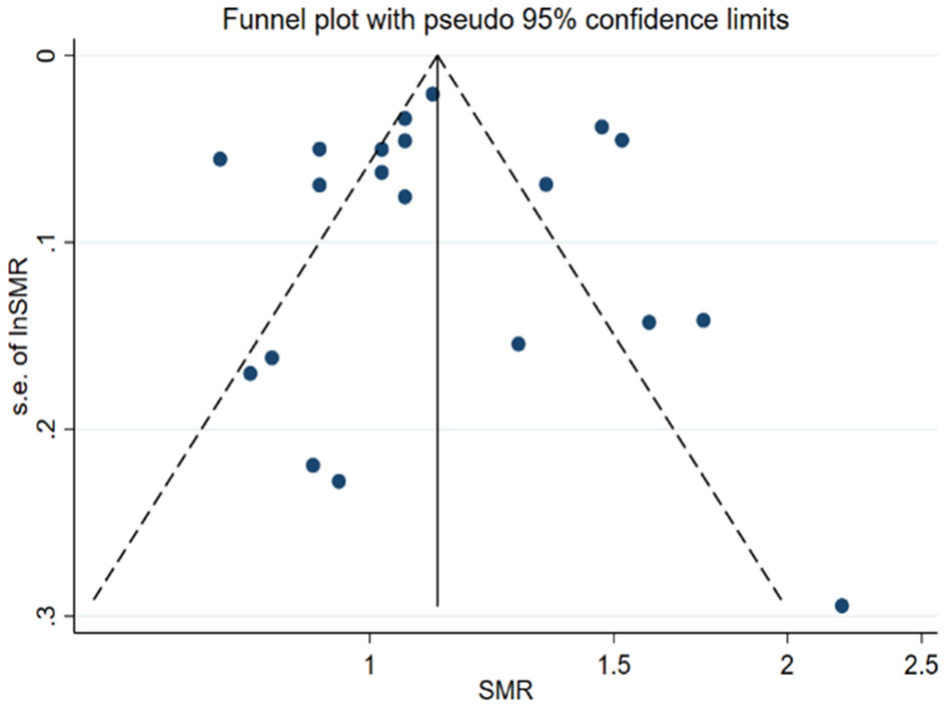
Funnel plot of 20 studies evaluating mortality of PsA patients compared with the general population.

Funnel plot symmetry analysis using both the Mantel–Haenszel (M–H) fixed-effect method and the Peto method demonstrated no detectable publication bias across comparative analyses (**Figure 3**). Statistical evaluation using Egger’s linear regression test (p=0.966) and Begg’s rank correlation test (p=0.966) further confirmed the absence of significant asymmetry in the distribution of effect estimates.

## Discussion

Substantial epidemiological evidence has established elevated mortality in several autoimmune conditions, notably systemic lupus erythematosus, Sjögren’s syndrome, and rheumatoid arthritis (29–31). However, the mortality risk associated with PsA remains a subject of debate. As a systemic autoimmune disorder, PsA involves multiple domains. Our meta-analysis revealed a 12% increase in mortality risk among patients with PsA compared to the general population.

Our analysis revealed a significantly elevated mortality risk in female patients with PsA relative to age-matched general population controls. A total of five studies reported that mortality or SMRs among female patients surpassed those of their male counterparts, indicating a potentially worse prognosis in women (5, 12, 17, 18, 25). However, this finding should be interpreted with caution due to the limited number of studies providing sex-specific SMRs. Further research is warranted.

Heterogeneity across studies was expected. Notably, compared to the general population, elevated mortality was observed among Asian patients with PsA, but not among patients in North America or Europe. This disparity could potentially be attributed to divergent treatment modalities, although the therapeutic strategies seemed similar across the studies.

We also noted a trend toward higher mortality risk in population-based studies compared with referral center cohorts. Additionally, the meta-analysis revealed elevated SMRs among studies involving over 1,000 patients. Given the publication of large-scale studies in recent years, this may explain the differences between our findings and those of earlier meta-analyses (13).

Our analysis demonstrated reduced overall life expectancy in patients with PsA compared to general population cohorts. Complications emerged as pivotal determinants contributing to the heightened mortality risk observed, particularly malignancy, cardiovascular and cerebrovascular diseases, and infection/respiratory disease, which were among the most frequently reported causes of death.

The association between PsA and cancer has been investigated in multiple studies. A systematic review and meta-analysis reported a significant association between psoriasis and overall cancer risk, but not between PsA and cancer (32). Conversely, a nationwide population-based cohort study in Korea found that individuals with PsA had a higher cancer risk than age- and sex-matched controls, especially for non-melanoma skin cancer (NMSC), lymphoma, and thyroid cancer (33). Similarly, a population-based study using the UK Clinical Practice Research Datalink reported an increased incidence of hematological cancers in patients with PsA (34). The chronic inflammatory nature of PsA may contribute to the elevated cancer risk by promoting the accumulation of somatic mutations and inducing various epigenetic changes during carcinogenesis (35). Lifestyle factors such as smoking and obesity—which are common risk factors for both PsA and cancer—may also play a role in increasing cancer risk among patients with PsA (36, 37). Notably, the use of biologic agents, including tumor necrosis factor inhibitors, has not been shown to increase cancer risk in PsA patients (32, 38). This calls for further investigation with longer follow-up and may serve as a reminder of the necessity of cancer screening in the management of PsA.

The elevated risk of cardiovascular disease (CVD) in PsA has been recognized for decades and has emerged as a major contributor to mortality, as evidenced by our findings and previous research (4, 17, 39). Chronic inflammation is known to consistently accelerate atherosclerosis, and systemic inflammatory markers have been strongly linked to cardiovascular events (40). Besides, as an important cytokine in the pathogenesis of PsA, IL-17 can contribute to the increased risk of cardiovascular events by affecting vascular and cardiac cells (41). Meng et al. demonstrated that systemic inflammatory markers independently increase the risk of major adverse cardiovascular events (MACE), underscoring the crucial role of inflammation in cardiovascular morbidity among PsA patients (42). Additionally, their research indicated that treatment with methotrexate and non-steroidal anti-inflammatory drugs (NSAIDs) was associated with a reduced risk of MACE, whereas the use of glucocorticoids appears to be harmful. Consequently, it is imperative to incorporate cardiovascular risk assessment in the therapeutic strategies for PsA patients.

In addition to malignancies and cardiovascular-related mortality, we also observed an increased mortality risk attributed to infections or respiratory diseases in patients with PsA. The expanding array of therapeutics available for PsA in recent years has prompted increased scrutiny regarding their safety profiles. Chiu et al. reported a heightened susceptibility to infections associated with targeted therapies compared to placebo in patients with PsA (43).

While previous studies have indicated that serious infections are rare among PsA patients treated with biologic or targeted disease-modifying antirheumatic drugs (DMARDs), it is noteworthy that most of these studies were based on randomized controlled trials, which are often characterized by strict participant selection criteria and relatively short follow-up durations (44, 45). Consequently, it is imperative to acknowledge the inherent limitations of such analyses in demonstrating an elevated risk of a rare side effect. Therefore, further real-world data are needed in the future.

Undoubtedly, several limitations of this meta-analysis warrant consideration. First, the included studies exhibited heterogeneity across multiple dimensions. Although most studies were of moderate to high quality, variability in study design and execution may have contributed to residual heterogeneity. We addressed this, to some extent, through prespecified subgroup analyses, sensitivity testing, and exclusion of an outlier study with a substantially elevated SMR. Importantly, the core finding of increased mortality risk in PsA patients relative to the general population remained consistent.

Second, the limited number of studies examining risk factors and causes of mortality warrants cautious interpretation of these findings. Finally, the lack of treatment-specific data across studies precluded any analysis of medication effects on disease progression and mortality outcomes.

## Conclusion

Our investigation revealed a 1.12-fold increase in mortality risk among patients with PsA compared to the general population, although the presence of heterogeneity warrants cautious interpretation. Predominant causes of mortality included malignancies, cardiovascular and cerebrovascular diseases, as well as infection/respiratory conditions. Additional population-based studies—particularly those from diverse geographical regions and with extended follow-up durations—are needed to further elucidate mortality risk and its determinants in PsA.

## Data Availability

The original contributions presented in the study are included in the article/**Supplementary Material**. Further inquiries can be directed to the corresponding author.
