# Supplementary material for: Mortality in patients with psoriatic arthritis: a systematic review and meta-analysis
Source: Front Immunol. 2025 Aug 18;16:1622159. doi: 10.3389/fimmu.2025.1622159 (PMC12399637; doi:10.3389/fimmu.2025.1622159)
Supplement: Supplementary file 1 [file DataSheet1.pdf]

Supplementary Files

Appendix S1. Protocol of systematic review and meta-analysis.

Table S1. Newcastle-Ottawa scale for quality assessment of included cohort studies.

Table S2. Predictors and causes of mortality of patients in the included studies

Figure S1. Funnel plot of mortality of female and male PsA patients compared with the general population

Figure S2. Funnel plot for subgroup analyses of region, setting and number of patients

Appendix S1. Protocol of systematic review and meta-analysis

Mortality in patients with psoriatic arthritis: A systematic review and meta-analysis

Aim

To investigate the mortality in patients with psoriatic arthritis (PsA) by conducting a meta-analysis of observational studies.

Population

Patients with PsA

Comparison

- The general population or matched control population, or directly reported the standardized mortality ratio (SMR).

Outcome

Primary outcome

- The primary outcome of the study was all-cause mortality expressed as SMR in PsA patients compared with the general population.

Secondary outcome

- Secondary analyses were performed according to different regions, settings, number of patients. The causes of death and risk factors for mortality were also analyzed.

Search strategies

Databases searched:

- PubMed, Embase, and Cochrane Library
- From database inception through June 2025
- Using the phrase (psoriatic arthritis) and (death or mortality or survival or fatality) in all databases.

- Other sources: scientific meetings (the American College of Rheumatology, the European League Against Rheumatism) were searched for unpublished completed studies
- PubMed: ("arthritis, psoriatic"[MeSH Terms] OR ("arthritis"[All Fields] AND "psoriatic"[All Fields]) OR "psoriatic arthritis"[All Fields] OR ("psoriatic"[All Fields] AND "arthritis"[All Fields])) AND ("death"[All Fields] OR "mortality"[All Fields] OR "survival"[All Fields] OR " fatality"[All Fields])
- EMBASE: ('psoriatic arthritis'/exp OR 'psoriatic arthritis' OR (psoriatic AND ('arthritis'/exp OR arthritis))) AND (death OR mortality OR survival OR fatality)
- Cochrane library: (psoriatic arthritis) and (death or mortality or survival or fatality)

The systematic search will be carried out by HH and WX. All references will be collated on Endnote X8. After removal of duplicates using the function on Endnote X8, the remaining articles will be subject to screening and review steps:

All studies included in any of the reviews deemed relevant were retrieved in full-text and judged according to the above eligibility criteria.

1. Screening: Title and abstract will be browsed with removal of non-relevant studies. This step will be done by two authors (HH and WX) individually. Non-relevant studies will be discarded at the reviewers' discretion. Specific reason for exclusion will not be recorded, and the reason will be given as "non-relevant".

2. Review: Remaining articles will be further judged in full-text by HH and WX independently, according to the following inclusion criteria. Reasons for exclusion will be recorded.

Inclusion criteria

1. a population-based or single center or multi-center cohort study,

2. reported all-cause mortality rate in PsA patients,

3. compared the mortality rate with the general population or matched control population, or directly reported the SMR.

Data extraction

Data was extracted using piloted forms on Word independently by both investigators (HH and FY). The following information will be extracted:

- Author name, publication year, region, study design, data source, enrolment period, follow-up time, number of PsA patients, sex composition, age, number of death events, sex composition in death events, SMR, comparison group, causes of deaths, risk factors for mortality.

Statistical analysis

All statistical analyses were conducted using Stata Statistical Software version 15.1. SMR with an accompanying 95% confidence interval (CI) of PsA patients compared with comparison group was calculated by DerSimonian & Laird random-effect method. Separate meta-SMRs in male and female patients were also calculated. Heterogeneity was quantitively evaluated by using the I^2 statistics and regarded as being low (≤25%), moderate (>25% and ≤50%), substantial (>50%). Sources of heterogeneity were further explored by subgroup meta-analyses and sensitivity analyses. All included studies were accordingly stratified by gender, study design (prospective or retrospective cohort), region, population setting (population-based vs. hospital/clinic-based samples), cohort type (inception vs. non-inception), patient number (more than or less than 1000), and follow-up period.

Sensitivity analysis

Sensitivity analysis was performed by deleting each individual study to evaluate the quality and confirm the consistency of the results.

Risk of bias assessment

The quality of individual studies was assessed using the Newcastle-Ottawa Scale by independent assessment (HH and YF) which has been widely used to assess the quality of nonrandomized studies such as cohort and case-control studies. Briefly, this scale allocates points for appropriateness

of participant selection (0-4 points), comparability (0-2 points) and exposure or outcome (0-3 points). A maximum of 9 points was assigned to each study and a final score ≥ 6 was regarded as high quality.

SELECTION

- Representativeness of the exposed cohort:

1: Given if representative of the average patient with PsA in the community.

0: Given if selected from a group of volunteers or derivation of the cohort is not described.

- Selection of the nonexposed cohort:

1: Given if drawn from the same community as the exposed cohort.

0: Given if selected from a group of volunteers or derivation of the cohort is not described.

- Ascertainment of exposure

1: Given if obtained by a secure record or structured interview.

0: Given if no description is given or self-report.

- Demonstration that outcome was not present at start of study:

1: Given if demonstrated.

0: Given if not demonstrated.

COMPARABILITY

- Comparability of cohorts on age/sex

1: Given if the answer is yes.

0: Given if the answer is no.

- Comparability of additional risk factors for mortality

1: Given if additional risk factors for mortality were comorbidities, lifestyle risk factors, etc.

0: Given if not demonstrated.

OUTCOME

- Assessment of outcome:

1: Given if obtained by independent blind assessment or record linkage.

0: Given if obtained from self-report or not described.

- Was follow-up long enough for outcomes to occur:

1: Given if follow-up was long than 5 years.

0: Given if follow-up was not long enough.

- Adequacy of follow-up of cohorts:

1: Given if complete follow-up is provided or ≥80% of follow-up is provided.

0: Given if follow-up rate was <80% or no description is provided.

Table S1. Newcastle-Ottawa scale for quality assessment of included cohort studies

Quality assessment criteria	Wilson FC et al., 2009	Buckley C et al., 2010	Ahlehoff O et al., 2011	Mok CC et al., 2011	Love TJ et al., 2013	Ogdie A et al., 2014	Juneblad K et al., 2016	Lee MS et al., 2017	Cheung TT et al., 2018	Dai XY et al., 2018	Skov L et al., 2019	Elalouf O et al., 2020	Colaco K et al., 2021	Karmacharya P et al., 2021	Bournia VK et al., 2021	Daddad A, et al., 2022	Iskandar IYK et al., 2022	Kerola AM et al., 2022	Erden A et al., 2023	Exarchou S et al., 2023
**Selection**																				
Representativeness of exposed (PsA) cohort?	*	-	*	*	*	*	-	*	-	*	*	-	*	*	*	*	*	*	-	*
Selection of the non-exposed cohort?	*	*	*	*	*	*	-	*	*	*	*	-	*	*	*	*	*	*	*	*
Ascertainment of exposure (PsA diagnosis)?	*	*	*	*	*	*	*	*	*	*	*	*	*	*	*	*	*	*	*	*
Demonstration that outcome of interest was not present at start of study?	*	*	*	*	*	*	*	*	*	*	*	*	*	*	*	*	*	*	*	*
**Comparability**																				
Study controls for age/sex?	*	*	*	*	*	*	*	*	*	*	*	*	*	*	*	*	*	*	*	*
Study controls for additional risk factors for mortality?	-	-	-	-	-	-	-	-	-	-	-	-	-	-	-	-	-	-	-	-
**Outcome**																				
Assessment of outcome?	*	*	*	*	*	*	*	*	*	*	*	*	*	*	*	*	*	*	*	*
Was follow-up long enough for outcome to occur?	*	*	*	*	-	*	*	*	*	*	*	*	*	*	*	*	*	*	*	*
Adequacy of follow-up of cohorts?	-	*	*	-	-	-	-	-	-	-	-	-	*	-	*	*	*	*	*	*
**Overall Quality Score (Maximum = 9)**	**7**	**7**	**8**	**7**	**6**	**7**	**5**	**7**	**6**	**7**	**7**	**5**	**8**	**7**	**8**	**8**	**8**	**8**	**7**	**8**

Table S2. Predictors and causes of mortality of patients in the included studies

No.	Author, year of publication	Death events, n	Risk factors for mortality	Causes of death
1	Buckley C et al., 2010	37		cardiovascular disease (14), diseases of the respiratory system (10), malignancy (5)
2	Mok CC et al., 2011	51		Infection (17), cancer (10), cardiovascular (10), cerebrovascular (2), gastrointestinal (1), hepatic (2), respiratory system (1), renal failure (1), poisoning/injury (2), other (3), unknown (2)
3	Juneblad K et al., 2016	44	Higher mean DAI, axial involvement	Circulatory system (21), malignant neoplasm (14), respiratory disease(1), others (8)
4	Cheung TT et al., 2018	187		CV mortality (34), infection (58), cancer (36)
5	Skov L et al., 2019	764		Neoplasms (507), diseases of the circulatory system(198)
6	Elalouf O et al., 2020	225	Elevated acute phase reactants, presence of heart disease and presence of cancer	Malignant neoplasm (61), acute myocardial infarction (32), pneumonia (14)
7	Colaco K et al., 2021	221		circulatory diseases (58), cancer (48), and respiratory diseases (28)
8	Haddad A, et al., 2022	471	Older age, male sex, lower socioeconomic status, increased BMI, increased Charlson comorbidity index scores, and history of psoriasis or hospitalization in 1 year prior to entry	Malignancy (122), ischemic heart disease (74), diabetes (29), cerebrovascular disease (26), septicemia (26)
9	Kerola AM et al., 2022	911		Circulatory system (25.6%), Ischaemic heart diseases (9.3%), Cerebrovascular diseases (4.0%), Neoplasms (33.6%)
10	Erden A et al., 2023	31	Male patients, older age, being less educated, higher acute phase reactants, metabolic comorbidities	Cardiac (15), infections (15), pulmonary (2), malignancy (9), renal 5), hepatobiliary (4), and other causes (5)
11	Exarchou S et al., 2023	3121	Lower socioeconomic status, prior joint surgery, presence of all assessed general comorbidities at the start of follow-up	Cardiovascular disease (905), diabetes mellitus (66), chronic kidney disease (12), infection (212), chronic pulmonary disease (159), malignancy (905), suicide (72), other (843)

Figure S1. Funnel plot of mortality of female (A) and male (B) PsA patients compared with the general population

A

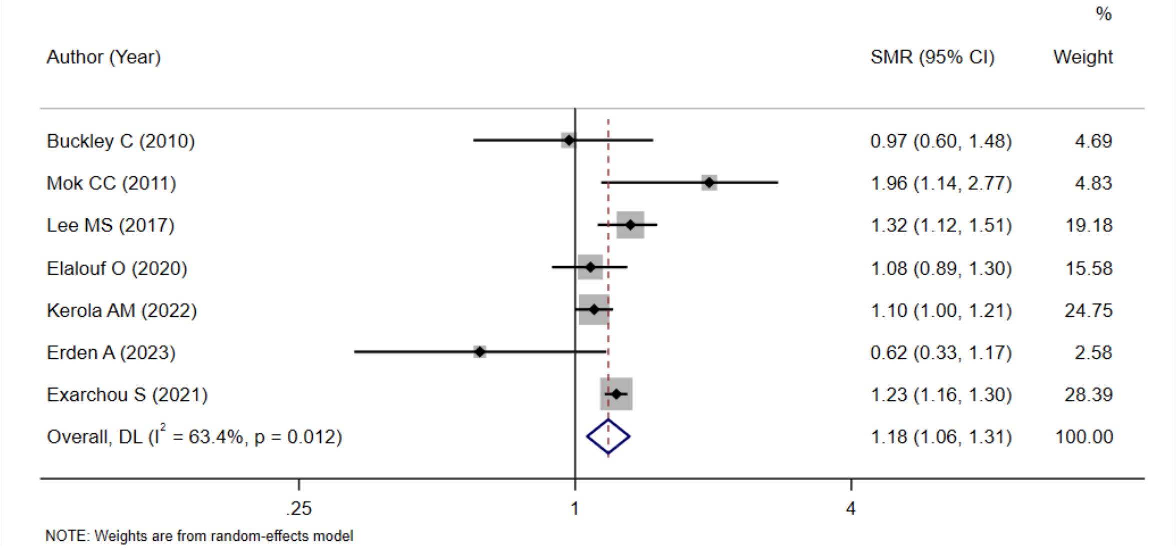

B

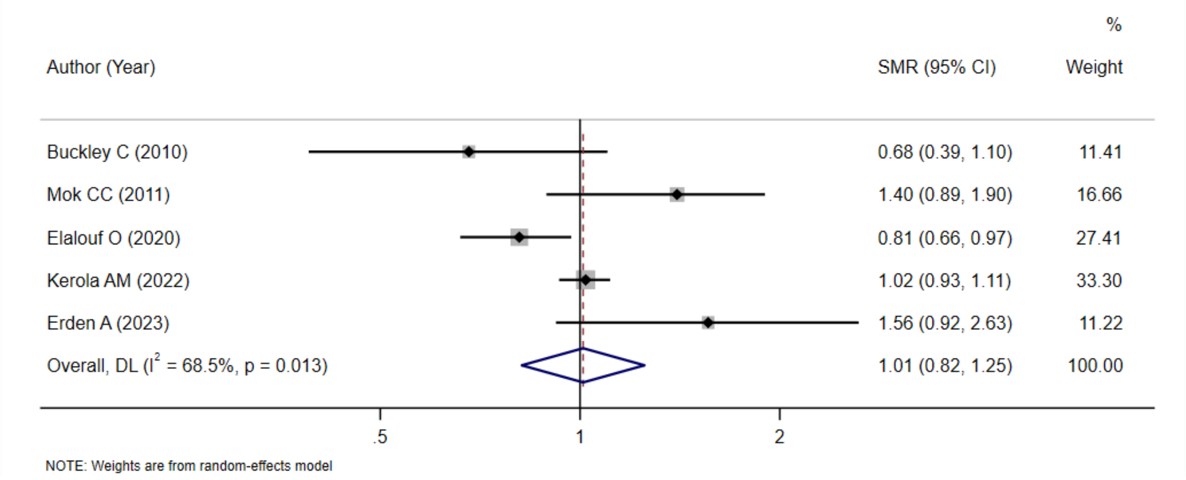

Figure S2. Funnel plot for subgroup analyses of region (A), setting (B) and number of patients (C)

A

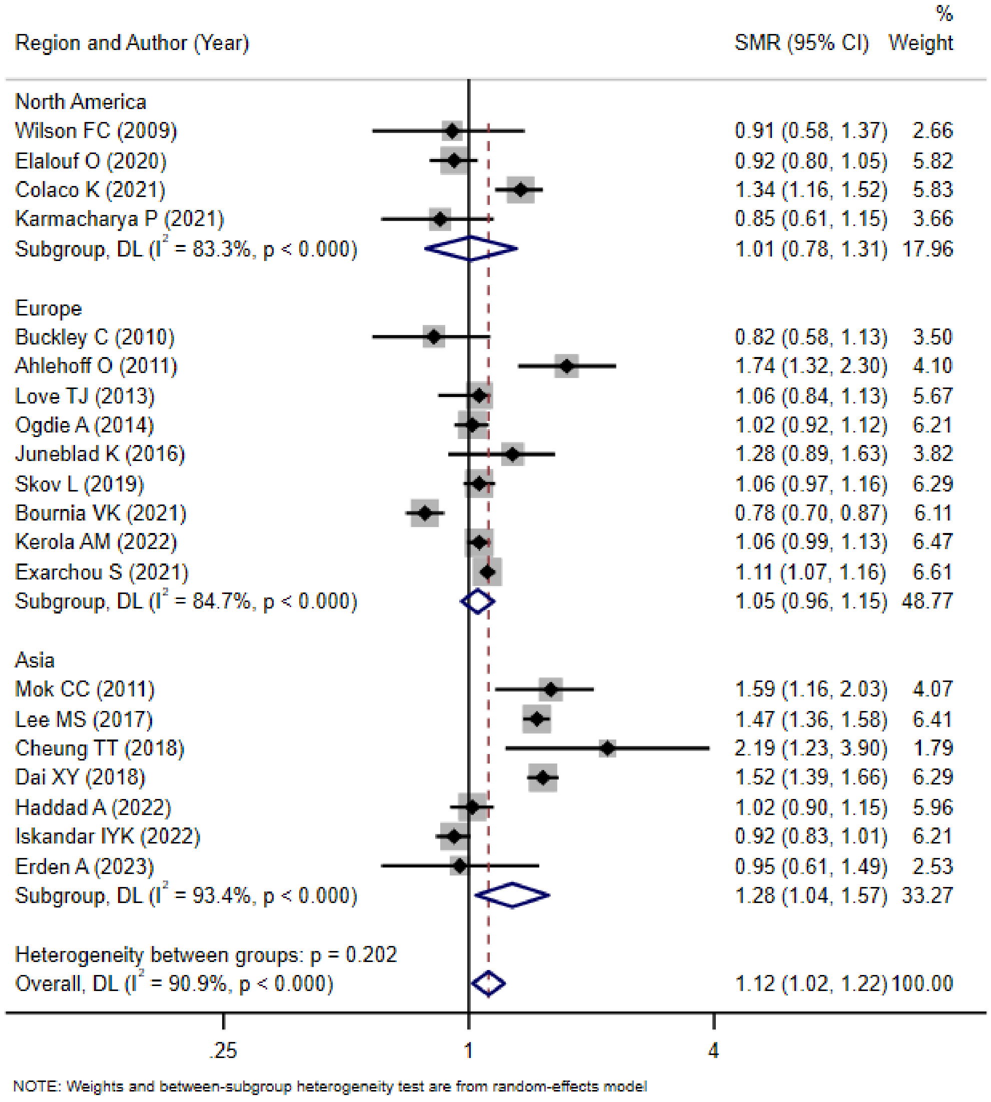

B

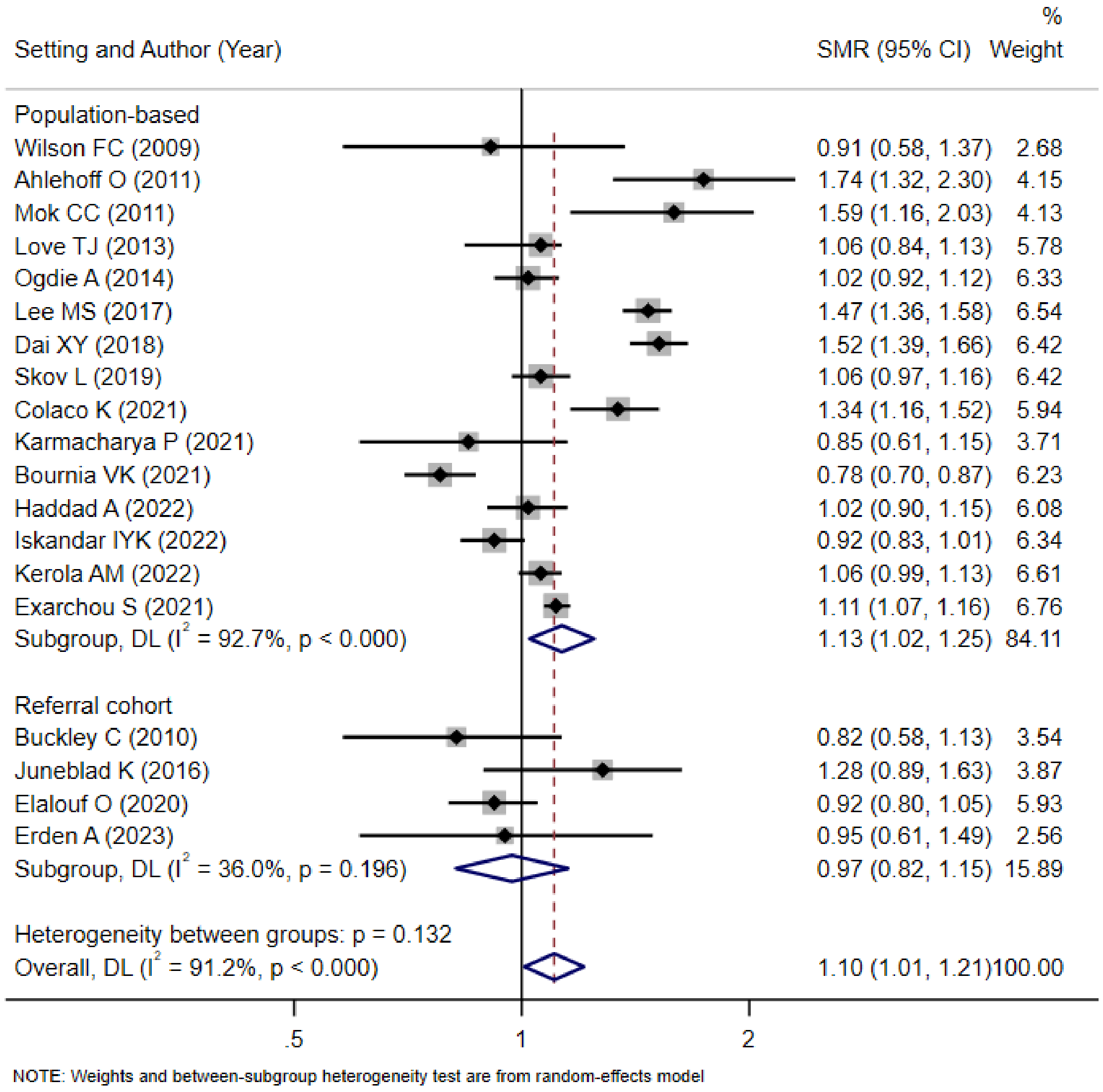

Setting and Author (Year)
SMR (95% CI)
% Weight
Population-based
Wilson FC (2009) 0.91 (0.58, 1.37) 2.68
Ahlehoff O (2011) 1.74 (1.32, 2.30) 4.15
Mok CC (2011) 1.59 (1.16, 2.03) 4.13
Love TJ (2013) 1.06 (0.84, 1.13) 5.78
Ogdie A (2014) 1.02 (0.92, 1.12) 6.33
Lee MS (2017) 1.47 (1.36, 1.58) 6.54
Dai XY (2018) 1.52 (1.39, 1.66) 6.42
Skov L (2019) 1.06 (0.97, 1.16) 6.42
Colaco K (2021) 1.34 (1.16, 1.52) 5.94
Karmacharya P (2021) 0.85 (0.61, 1.15) 3.71
Bournia VK (2021) 0.78 (0.70, 0.87) 6.23
Haddad A (2022) 1.02 (0.90, 1.15) 6.08
Iskandar IYK (2022) 0.92 (0.83, 1.01) 6.34
Kerola AM (2022) 1.06 (0.99, 1.13) 6.61
Exarchou S (2021) 1.11 (1.07, 1.16) 6.76
Subgroup, DL (I² = 92.7%, p < 0.000) 1.13 (1.02, 1.25) 84.11
Referral cohort
Buckley C (2010) 0.82 (0.58, 1.13) 3.54
Juneblad K (2016) 1.28 (0.89, 1.63) 3.87
Elalouf O (2020) 0.92 (0.80, 1.05) 5.93
Erden A (2023) 0.95 (0.61, 1.49) 2.56
Subgroup, DL (I² = 36.0%, p = 0.196) 0.97 (0.82, 1.15) 15.89
Heterogeneity between groups: p = 0.132
Overall, DL (I² = 91.2%, p < 0.000) 1.10 (1.01, 1.21) 100.00
.5
1
2
NOTE: Weights and between-subgroup heterogeneity test are from random-effects model

C

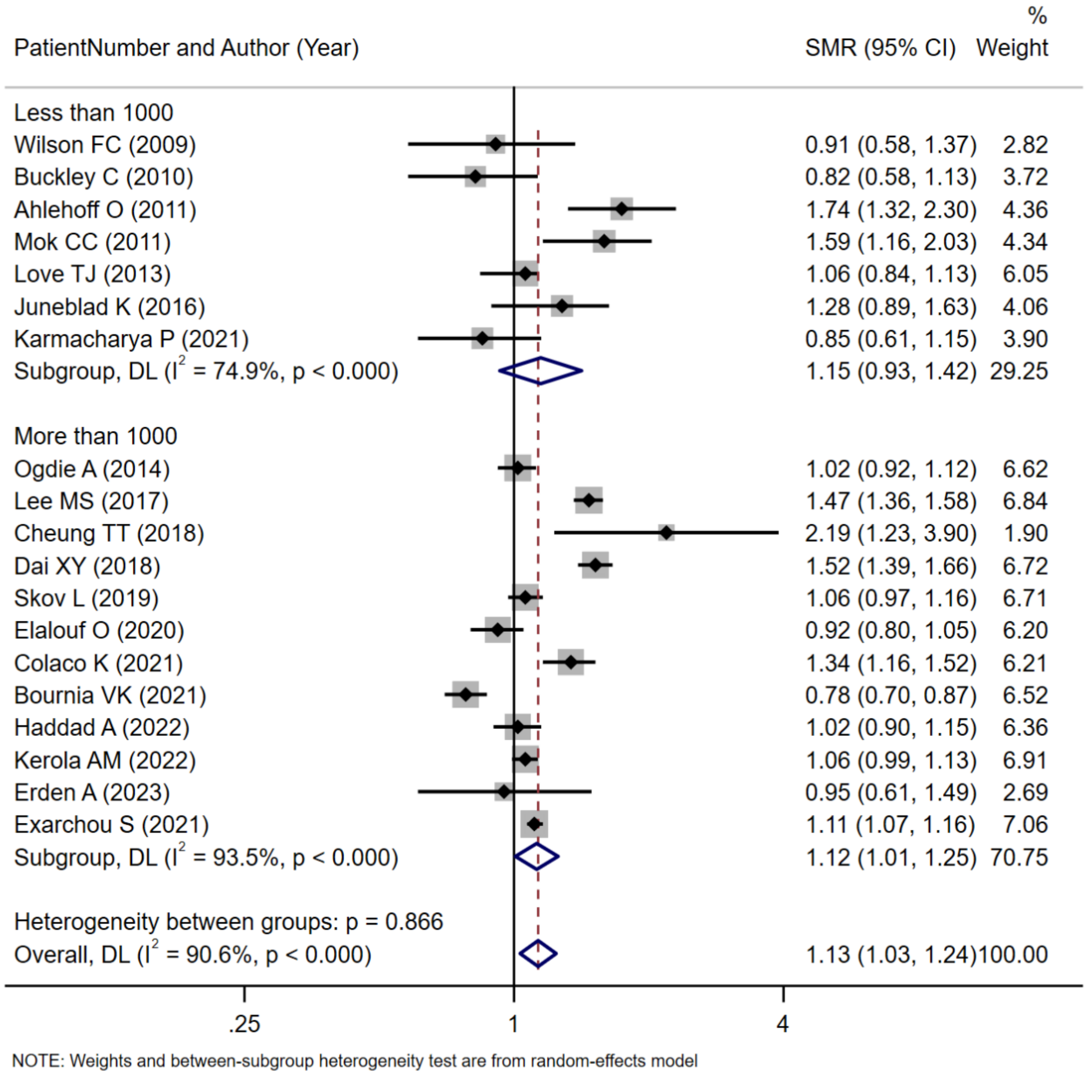
PatientNumber and Author (Year)
SMR (95% CI)
% Weight
Less than 1000
Wilson FC (2009) 0.91 (0.58, 1.37) 2.82
Buckley C (2010) 0.82 (0.58, 1.13) 3.72
Ahlehoff O (2011) 1.74 (1.32, 2.30) 4.36
Mok CC (2011) 1.59 (1.16, 2.03) 4.34
Love TJ (2013) 1.06 (0.84, 1.13) 6.05
Juneblad K (2016) 1.28 (0.89, 1.63) 4.06
Karmacharya P (2021) 0.85 (0.61, 1.15) 3.90
Subgroup, DL (I^2 = 74.9%, p < 0.000) 1.15 (0.93, 1.42) 29.25
More than 1000
Ogdie A (2014) 1.02 (0.92, 1.12) 6.62
Lee MS (2017) 1.47 (1.36, 1.58) 6.84
Cheung TT (2018) 2.19 (1.23, 3.90) 1.90
Dai XY (2018) 1.52 (1.39, 1.66) 6.72
Skov L (2019) 1.06 (0.97, 1.16) 6.71
Elalouf O (2020) 0.92 (0.80, 1.05) 6.20
Colaco K (2021) 1.34 (1.16, 1.52) 6.21
Bournia VK (2021) 0.78 (0.70, 0.87) 6.52
Haddad A (2022) 1.02 (0.90, 1.15) 6.36
Kerola AM (2022) 1.06 (0.99, 1.13) 6.91
Erden A (2023) 0.95 (0.61, 1.49) 2.69
Exarchou S (2021) 1.11 (1.07, 1.16) 7.06
Subgroup, DL (I^2 = 93.5%, p < 0.000) 1.12 (1.01, 1.25) 70.75
Heterogeneity between groups: p = 0.866
Overall, DL (I^2 = 90.6%, p < 0.000) 1.13 (1.03, 1.24)100.00
.25
1
4
NOTE: Weights and between-subgroup heterogeneity test are from random-effects model
